# Sensor-Data Fusion for Multi-Person Indoor Location Estimation

**DOI:** 10.3390/s17102377

**Published:** 2017-10-18

**Authors:** Parisa Mohebbi, Eleni Stroulia, Ioanis Nikolaidis

**Affiliations:** Department of Computing Science, University of Alberta, Edmonton, AB T6G 2R3, Canada

**Keywords:** indoor localization, activities of daily living, activity recognition, sensor fusion, passive infrared (PIR) sensors, Bluetooth Low-Energy (BLE), BLE beacons, Estimote, anonymous sensing, eponymous sensing

## Abstract

We consider the problem of estimating the location of people as they move and work in indoor environments. More specifically, we focus on the scenario where one of the persons of interest is unable or unwilling to carry a smartphone, or any other “wearable” device, which frequently arises in caregiver/cared-for situations. We consider the case of indoor spaces populated with anonymous binary sensors (Passive Infrared motion sensors) and eponymous wearable sensors (smartphones interacting with Estimote beacons), and we propose a solution to the resulting sensor-fusion problem. Using a data set with sensor readings collected from one-person and two-person sessions engaged in a variety of activities of daily living, we investigate the relative merits of relying solely on anonymous sensors, solely on eponymous sensors, or on their combination. We examine how the lack of synchronization across different sensing sources impacts the quality of location estimates, and discuss how it could be mitigated without resorting to device-level mechanisms. Finally, we examine the trade-off between the sensors’ coverage of the monitored space and the quality of the location estimates.

## 1. Introduction

According to a 2012 study commissioned by the Alzheimer’s Society of Canada, 747,000 Canadians have some type of cognitive impairment, including dementia, and this number is expected to double by 2031. People with dementia experience challenges with daily activities (e.g., cooking meals, ironing, taking medication, personal care), such as misplacing materials and failing to complete tasks in the right sequence. Having accurate information about an older adult’s daily activities, and the patterns of these activities, can provide rich information on his/her abilities and capacity for functional independence. Major deviations in daily patterns should likely be considered as indicators of a person’s physical, cognitive and/or mental decline. Having such information could alert caregivers of potentially risky events and the need for additional support.

The advancing wave of Internet-of-Things technologies holds immense promise for enabling such data collection and analysis and for delivering appropriate support. In the $SmartCondo^{\mathrm{TM}}$ project, we have been developing a sensor-based platform for non-intrusively monitoring people at home, analyzing the collected data to extract information about the occupants’ activities, simulating the extracted information in a 3D virtual world, and generating recommendations—for themselves and their caregivers. To meet the non-obtrusiveness requirement of our platform, we have excluded from $SmartCondo^{\mathrm{TM}}$ any image and video capture devices. Of course, for the sake of reconstructing the ground truth via manual annotation, the experiments we carried out also included video cameras. However, in a production-environment deployment, no cameras would be used.

This hardware–software platform has been installed in the $SmartCondo^{\mathrm{TM}}$ simulation space—a dedicated teaching-and-research space in the University of Alberta’s Edmonton Clinic Health Academy (ECHA). The $SmartCondo^{\mathrm{TM}}$ is a fully functional apartment with one bedroom, a bathroom, and an open kitchen-and-living space. Infused into the apartment and its furnishings are sensors that record a variety of environmental variables (i.e., levels of light and sound, temperature, and humidity) as well as the activities of the occupant(s) (i.e., their motion and use of furniture, cabinetry, and appliances). The data acquired by these sensors is transmitted and analyzed into a central cloud-based repository. The $SmartCondo^{\mathrm{TM}}$ has recently been redesigned to include Bluetooth Low-Energy (BLE) beacons attached to different objects in the apartment. The occupants can be provided with a smartphone, running a background service that collects, and transmits to the $SmartCondo^{\mathrm{TM}}$ platform, signal-strength measurements from any nearby BLE beacons. These two types of data sources—sensors and beacons—are used to infer the occupants’ locations at each point in time. The server generates textual reports, spatial visualizations, and 3D virtual-world simulations for the inferred movements and activities of every occupant. In addition, it can potentially generate alerts for special incidents, which can be sent to the occupants’ caregivers, or anyone of their choice.

In our previous work, we investigated the trade-offs between the accuracy of the location-estimation process for one occupant, based on PIR (Pyroelectric or “Passive” Infrared) sensors only vs. the overall cost of the sensor installation [1]. Next, we studied how the use of RFIDs in addition to PIRs could be exploited to enable location recognition for multiple occupants [2]. In this paper, we report the results of our recent study on the relative effectiveness of motion sensors and BLE beacons for accurate location estimation for multiple occupants.

Multi-person location estimation, as a first step to activity recognition, is a challenging problem and has received relatively little attention in the literature. This is partly due to the implicit assumption that if the subjects carry with them active (hence, eponymous) devices, each person can be localized independently, in isolation of the rest; hence, any method for estimating the location of a single individual is assumed to generalize to multiple individuals. However, the situation is drastically different when one (or more) of the subjects do not systematically carry/wear such a device, either because they cannot, they do not want to, or they simply forget to—typical of many care-delivery scenarios. Estimating the location of an individual does not yield the same results when applied to a scenario when the individual is alone vs. when the individual is one among many in the observed space. For example, the radio frequency (RF) propagation environment in a given space varies over time because of the dynamics of human and object placement within that space. In fact, [3] has utilized the impact of humans on the RF environment to estimate the locations of multiple subjects, based on models of how the fingerprints of radio signal strength indicators (RSSIs) change in the presence of people in the space. Nevertheless, this method requires a large set of transmitters and receivers to cover the entire area in each room and the placement of the transmitters/receivers needs to be exact to ensure that they are in line of sight (LoS). We address more of the related work in the next section, noting that our assumptions align closer with those of [4] where individuals carry smartphones, with the notable difference that we allow one of the individuals to not wear or carry any identifying device.

Our own previous work on this problem [2] targeted the development of a method using RFID readers embedded in the environment and wearable passive RFID tags. Such an approach is limited in terms of practicality because RFID readers today—especially if endowed with large antennas to attain reasonable range—are difficult to embed in everyday surroundings without expensive retrofitting of the space (and frequently violating the aesthetics of “home” environments). The passive RFID tags also have to be embedded in everyday items (e.g., clothing), and hence the reliability is generally compromised unless specially treated to cope with washing and other everyday wear-and-tear.

In this paper we focus on (a) the fusion of data collected by PIR motion sensors with data collected from tiny BLE beacons attached with simple adhesive glue on surfaces around the home and collected through an application running on the occupants’ Android smartphones. We then (b) evaluate the effectiveness of our method through an empirical study in the $SmartCondo^{\mathrm{TM}}$, exploring caregiver scenarios where one individual does not wear a device nor carries a smartphone, while the second (typically the caregiver) carries such a device. In addition to its applicability to realistic care-giving scenarios, the main advantage of the technique described here is that the location of the two individuals can be accurately determined.

The rest of this paper is organized as follows. Section 2 places our work in the context of the most recent related work in the field of localization and activity recognition. Section 3 shows the architecture of our system and our location estimation method. Section 4 outlines our experimental methodology and results. At the end, Section 5 concludes the paper with a summary of our findings.

## 2. Related Work

Over the past decade, the area of indoor location estimation has resulted in many proposals and research findings, with varying degrees of applicability in real environments. One strategy to solving this problem is based on RSSI fingerprinting. RSSI readings for specific points in the space are collected in a database of readings at known locations; at run time, when a new RSSI reading is received, it is compared to the dataset and the “nearest” point (according to some definition of distance) is selected as the likely location. For example, [5] introduced a fingerprinting system utilizing passive RFID tags and four RFID readers and used a k-nearest-neighbor (kNN) method to select the nearest points to the received signal when localizing. In a building with two bedrooms and with the space logically divided in a grid-like fashion of cells of size  1.5×1.5m${}^{2}$, their method achieved a reported accuracy as 96% when localizing at the granularity of a grid cell. Typical of fingerprinting methods, it requires prior RSSI data collection, which is a task sensitive to the environment that needs to be repeated should the environment change in ways that impact the radio frequency propagation (e.g., when furniture is added/removed/moved).

Another school of thought pays attention to the kinematics and relates the generation of the location estimates with the direction of movement of the individuals. An example is the pedestrian dead-reckoning (PDR) algorithm [6], where new location estimates are based on previous known locations. For example, in [7], a PDR was proposed with WiFi and iBeacon signals, used to calibrate the drifting of the PDR algorithm by converting their RSS values to meters via a path-loss model. This family of methods requires a fall-back scheme for estimating locations in the absence of previous estimates in two cases: (a) when the initial location needs to be established, and (b) when sufficient error has accumulated, based on the estimates following the kinematics, such that a “re-initialization” of the estimation needs to take place. While we take some measures to consider the kinematic behavior of the individuals, we do not rely on it, as the activities in which an individual is engaged in a small indoor space call for frequent changes of direction and speed, and some tasks are fundamentally unsuitable for dead-reckoning approaches (e.g., broom sweeping). In another PDR approach in [8], the authors used WiFi fingerprints to calibrate the PDR error after time, and they performed their experiments when the subject was walking in a path and used the location estimation approach to track the subject. In [9], an RFID-based indoor location estimation is proposed for the elderly living alone, which uses both RSSI for localizing the subject and fuses it with a PDR using accelerometer data to step and direction detection to increase the accuracy.

We hasten to add that in the IPIN 2016 offline competition [10], the best team introduced a PDR with RSS fingerprinting for the initial position with accuracy of 2 m for a single individual in one of the spaces considered. The other four best teams performed RSSI fingerprinting, PDR, and MAC address filtering and fingerprinting with less accurate results.

In [11], PDR is used with WiFi and iBeacon fingerprinting: the iBeacon is used only where the WiFi signal is not strong enough. Similar to profiling, methods that use path-loss models rely on a model-configuration step, specific to the building and the environment where they are deployed, and changes to the environment require a re-computation of the path-loss model parameters to preserve accuracy.

The approach we discuss in this paper considers only RSSI values higher (stronger) than −70 dBm. The choice for this threshold comes from our previous work [12], and reflects situations where the subject is very close (approximately within one meter) of the Estimote beacons we use. In this fashion, the RSSI values only matter when they are strong enough to act as a proximity sensors rather than as a model for distance calculation.

A self-calibrated system is proposed in [13], where the sensors (smartphones in this case) communicate with each other to determine first their locations relative to each other, and subsequently the location of the target wearing one of these transmitters/receivers. A master node sends acoustic signals to the others to localize themselves with respect to the master node with the power and the direction of arrival (DOA) of signals received by the two microphones on the smartphone. An iterative expectation-maximization method is then used for the nodes to communicate their local estimate to the rest of the nodes. While the reported results appear to be excellent, they are produced under a completely static network—an assumption incompatible with most realistic scenarios. Static nodes are also used in the evaluation of the method outlined in [14], which utilizes a trilateration algorithm to localize each node in a multi-sensor network after converting the received signal strengths to meters via a path-loss model. An interesting feature of this algorithm is that it incorporates a means to temporally align the collected signal strength readings. In [15], an anchor-free self-calibration is proposed by means of an “Iterative Cole Alignment” algorithm based on a spring-mass simulation [16], and ensures synchronization by assuming that all receiving devices are linked to the same laptop; this method was evaluated assuming that the target always remains within a confined area.

The general question of how localization methods are evaluated arises in many publications, including the ones we discussed above. For example, static node configurations and artificially confined locations for the targets are fundamentally unrealistic and are bound to fail in the scenarios motivating our research. In this study, we collect sensor data resulting from the movement of one (or two) individual(s) in an actual apartment, following real (albeit scripted for the sake of consistency) movement scenarios throughout this apartment.

Indeed, when trying to use data collected from multiple sensors for location estimation (and activity recognition), a noticeable problem is sensor synchronization: most of the time, the clocks of the emitting sensors and devices involved are not completely synchronized. The approach proposed in [17] assumes that multiple sensors—each with its own clock and all connected to a single host—collect their timestamped observations in a FIFO (First-In-First-Out) structure; the host fetches the sensor data and performs a reconstruction of the sensor sample times, assuming a constant drift for each sensor and deterministic communication times. In our work, synchronization is not explicitly solved at the data-collection step; instead, we introduce the concept of a time “window” which abstracts the timestamp units at a coarser granularity and allows our method to ignore the imperfect synchronization of the data sources/sensors. As we will see, the window size can have a substantial effect on the accuracy of results.

The field of multiple person location estimation has received less attention from researchers. The majority of work in this area has been limited to counting how many occupants there are in the space. For example, [18] uses only binary sensors to count the people present within an area, eliminating the outliers due to incorrect sensor firing. The algorithm is initialized with the assumption that only the minimum number of sensors are outliers, and repeatedly increases the number of outliers until a solution is produced. Unfortunately, this method cannot recognize two occupants when their movement paths cross. [19] uses RFID tags and readers for the same person-counting task: the method maintains an uncertainty circle for each person, with a radius computed as the product of their speed of movement multiplied by the time lapsed; when a new event comes from a reader, the method assumes the person most likely to have moved in the vicinity of the reader based on their uncertainty circle and their direction of movement. A more recent paper by the same group [20] uses a much more expensive Kinect sensor to actually estimate the occupants’ locations.

When using motion sensors, it is important to know how to place them to achieve the best accuracy while minimizing the cost. [21] proposes a method for optimizing the placement of PIR sensors in order to meet the desired accuracy requirements while minimizing the cost. Their procedure hierarchically divides the space to sub-areas, based on walls and other obstacles such as large static furniture pieces. It then superimposes a grid on the space, whose length is determined by the accuracy needed, and solves the optimization problem of placing sensors so that the maximum possible number of grids cells are covered. In our group’s previous work, we developed a sensor-placement method that optimizes the information gain obtained by each additional PIR sensor placed in the space [1].

## 3. The Location-Estimation Method

**Figure 1:**
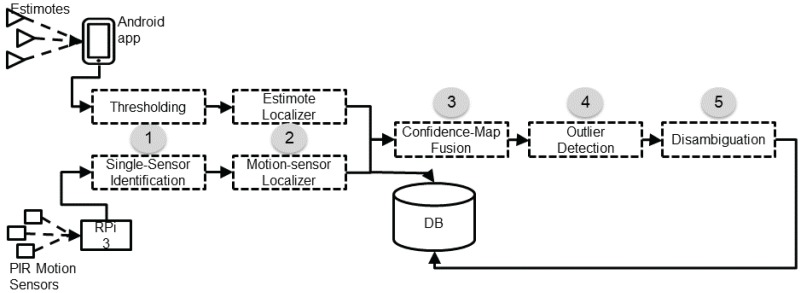
Structure of the proposed system. PIR: Pyroelectric (“Passive”) Infrared. DB: Data Base. RPi: Raspberry Pi 3.

Figure 1 provides the logical view of the $SmartCondo^{\mathrm{TM}}$ system architecture. The diagram depicts the two independent sensor-data collection paths combined at the server. Note that the architecture could technically admit more such independent simultaneously operating sensor feeds. The upper-left branch (Estimotes) captures eponymous data collection carried out by the smartphone device(s), and, in the future, by wearable devices. Estimote beacons are attached to objects in the surrounding space, with a considerable number of them attached to static objects (e.g., walls), or objects with trajectories known in advance (e.g., doors). Estimote “stickers” are fairly small and do not greatly impact the look-and-feel of the space; their interesting shapes and colors could even allow them to be perceived as decorative elements. The collection of data (RSSI values) is performed by Android devices running a special-purpose application which is aware of all installed stickers and their locations. When the device (smartphone or wearable), comes to the vicinity of any of these stickers, the application recognizes their presence and collects information about their RSSI and accelerometer signals. The RSSI is reported in dBm, ranging from −26 to −100 dBm when the transmitting power is set to a maximum of +4 dBm. The Android application streams this data to the $SmartCondo^{\mathrm{TM}}$ server every second. The format of the data sent from the Android device to the server is $<t_{i},device_{ID},beacon_{ID},RSSI>$, implying that at the specific timestamp $t_{i}$, the person carrying the device with ID=$device_{ID}$ received a transmission with a strength of RSSI from the Estimote with $beacon_{ID}$. Henceforth, we are using the terms $device_{ID}$ and $p_{ID}$ interchangeably.

The lower branch captures the anonymous sensing carried out by the PIR spot-type motion sensors placed on the ceiling. The PIRs can detect any movement within their sensing area which is a diamond-shape area, with the two diagonals equal to approximately 1.76 and 1.97 m. Groups of up to three motion sensors are connected via wires to a nearby wireless node, running a purpose-built firmware on a Texas Instruments MSP430 microcontroller using TI’s proprietary wireless module (CC1100). These nodes operate in the 900 MHz band, thus avoiding the heavily utilized 2.4 GHz band. The nodes wirelessly transmit the sensor observations to a Raspberry Pi 3 (RPi 3) with Internet access, which in turn uploads the data to a cloud-based server every three seconds. The format of the data uploaded by the RPi 3 to the server is $<t_{i},data>$, where the “data” element is a bitmap equal in length to the number of motion sensors installed. A 1 (0) at the $i_{th}$ position of the data bitmap implies that the sensor, corresponding to the $i_{th}$ index, detected (did not detect) movement within its corresponding sensing area. From a practical perspective, we should mention that in our three installations to date we have been able to hide the wires and the nodes inside ceiling tiles and behind cabinets, in order to minimize their impact on the aesthetics of the home.

It is important to note here some interesting similarities and differences between the two types of sensor data. Both types of data elements are timestamped with the time of the emitting device: the Android smartphone in the case of Estimotes, and the Raspberry Pi in the case of the motion sensors. Both include a payload: the $<beacon_{ID},RSSI>$ tuple in the case of Estimotes, and the data element in the case of the motion sensors. Note that the former includes information about a single beacon while the latter composes information about all the deployed motion sensors encoded in a bitmap. The most interesting difference between the two is the fact that Estimote data-transmission events are eponymous: each event includes the ID of a person, $p_{ID}$, (carrying the corresponding device, $device_{ID}$) perceived by the firing $beacon_{ID}$. This important difference characterizes motion sensors as anonymous and Estimotes as eponymous.

Our localization method involves five steps, diagrammatically depicted as a processing pipeline in Figure 1. The first two are specific to each type of sensor, and focus on data pre-processing and the generation of a first location estimate based only on the sensor(s) of this type. Should a new sensor type be integrated in our infrastructure, a corresponding step sequence would have to be developed specifically for this new sensor type. The remaining three steps are general and focus on fusing the sensor-specific location estimates.

### 3.1. Data-Stream Pre-Processing

As shown in Figure 1, all “raw” data is pushed to the database. A first, pre-processing step is applied on the raw data and the results are also stored in the database. As we have already discussed, each type of data is pre-processed differently. *RSSI thresholding* is applied to the Estimote data stream: a minimum threshold of −70 dBm is used to select the RSSI readings that are “significant” enough to be used for location estimation. This specific threshold value was motivated by experiments reported in [12]: roughly speaking, −70 dBm (or higher) RSSI strength suggests that the device is within approximately one meter of the transmitting beacon. In this fashion, the RSSI sensing effectively becomes an eponymous *proximity* sensor. The motion-sensor bitmaps are pre-processed to separate the individual motion sensor events. An additional pre-processing step is applied at this stage to add information helpful to the semantics of subsequent activity recognition, such as to label certain events as related to specific “actions” (e.g., an Estimote beacon attached to a kettle is associated with the action of “cooking”).

**Figure 2:**
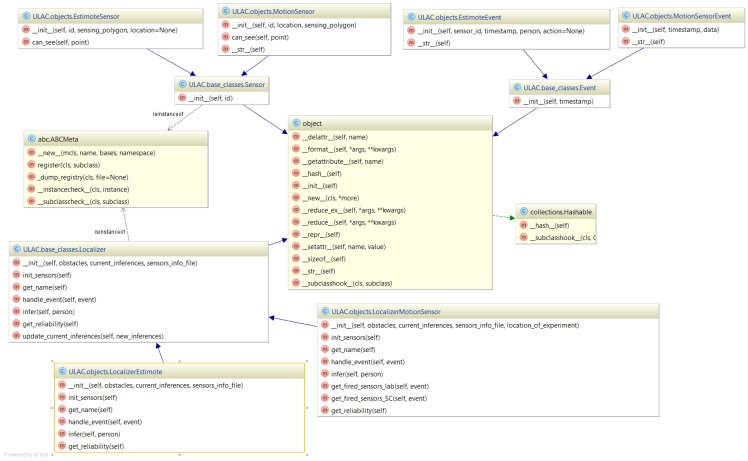
UML (Unified Modeling Language) diagram of the basic objects.

The pre-processed data streams are then fed to the type-specific localizers. We recognize that given the various technologies that might co-exist, a general format for the localizers needs to be defined to address current and future demands, and yet be able to integrate into the system easily. To this end, Figure 2 shows the UML (Unified Modeling Language) design of the sensor, event, and localizer classes, described in the following subsections. Figure 2 shows the base Localizer class with abstract methods for initializing its sensors and handling their corresponding incoming events. These methods are implemented in the children classes (i.e., the MotionLocalizer and EstimoteLocalizer), since each sensor type demands a different localization algorithm. The Sensor class has an ID which is a common field for every sensor. In addition, the EstimoteSensor and MotionSensor classes have an associated location where they are installed, and a sensing_polygon defining the area within which they perceive movement. They implement the can_see method, which decides whether a particular location is covered by their sensing area. The MotionEvent and EstimoteEvent classes are children of the Event class, and have a timestamp property. In the future, to add a new sensor type, *X*, to the system, one would first have to add a set of corresponding XEvent, XSensor, and XLocalizer classes, inheriting from Event, Sensor, and Localizer, respectively. The new XLocalizer class would also have to be added to the list of a system’s localizers to process incoming XSensor events and feed a corresponding output location estimate to the subsequent fusion steps.

### 3.2. Sensor-Specific Localizers

For the $SmartCondo^{\mathrm{TM}}$ platform, we have adopted the methodological assumption that individual localizers should be developed to work with each specific data stream producing a *location estimate*. Our location-estimate synthesis algorithm is general enough to take into account any number of such estimates and synthesize them into an overall location estimate. This design decision enables the extendibility of our platform: a new sensor-data stream implies the need for a new localization algorithm whose output is a location estimate that can be fed into the existing synthesis step. In this study, an Estimote-based Localizer and a Motion-Sensor-based (anonymous) Localizer are described.

As shown in Figure 2, every localizer should implement the same set of behaviors, including (a) initialization of the sensors whose data it consumes and (b) handling a data-transmission event. The key characteristic shared by all localizers is that their output should be described as a function of a “confidence” (or probability) metric over the space, which is represented as a two-dimensional grid of locations. That is, each localizer $L_{x}$ produces as output a $<t_{i},m_{xy}^{\left(L_{x}\right)},p_{ID}>$ tuple. The $m_{xy}^{\left(L_{x}\right)}$ is a positive metric (the larger the value, the more confident the localizer is about its estimate) that the individual $p_{ID}$ is at location (x,y) at time $t_{i}$. If a localizer is anonymous, the output is independent of $p_{ID}$ (i.e., the same for any candidate $p_{ID}$).

**Figure 3:**
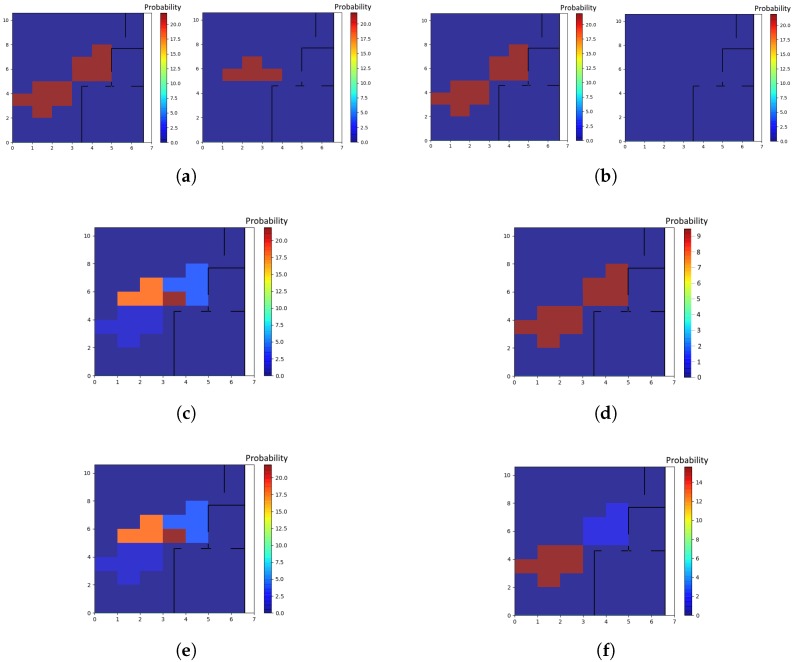
Confidence maps for 2 persons with motion sensors, and Estimote events for person 1 only. Figure 3a,c,e correspond to participant 1, and Figure 3b,d,f correspond to participant 2. (**a**) Initial confidence maps produced by coming out of the motion localizer (**right**) and estimote localizer (**left**) for participant 1; (**b**) Initial confidence maps produced by coming out of the motion localizer (**right**) and estimote localizer (**left**) for participant 2; (**c**) Confidence map for participant 1 after Fusion; (**d**) Confidence map for participant 2 after Fusion; (**e**) Final confidence map for participant 1; (**f**) Final confidence map for participant 2.

Algorithm 1 describes how each new event is processed upon arrival. The term “confMap” *confidence map* refers to the location estimates produced by each individual localizer, and by the subsequent fusion step. A number of *confidence maps* are illustrated in Figure 3. A localizer relying on anonymous sensors (e.g., motion sensors) produces a single confidence map, which is akin to a heat map of the space, with the color of each location corresponding to the localizer’s confidence that “some” occupant—any one of the known occupants in the space—is in that location. A localizer relying on eponymous sensors (e.g., Estimotes) produces a set of confidence maps, each one corresponding to an individual occupant, with a specific $p_{ID}$.

**Algorithm 1** Incoming event processing.1:**procedure** SensorFireHandler($e_{i}$)2:     **if** $e_{i}.type=ESTIMOTE$ **then**3:           **if** $e_{i}.RSSI>-70$ **then**            ▹ //Pre-processing part for Estimote events4:                ${\mathrm{confMap}}_{t_{i},p_{ID}}^{\left(L_{est}\right)}=\mathrm{EstimoteLocalizer}\left(e_{i,p_{ID}}^{\left(L_{est}\right)}\right)$5:                **for** $p_{ID}$ in P **do**                 ▹ //P is a set of all persons in system6:                     $motionMap={\mathrm{confMap}}_{t_{i-1},p_{ID}}^{\left(L_{ms}\right)}$          ▹ //Most recent confMap from $L_{ms}$7:                     **if** $e_{i}.person=p$ **then**8:                           $estimoteMap={\mathrm{confMap}}_{t_{i},p_{ID}}^{\left(L_{est}\right)}$9:                     **else**10:                         $estimoteMap={\mathrm{confMap}}_{t_{i-1},p_{ID}}^{\left(L_{est}\right)}$        ▹ //Most recent confMap from $L_{est}$11:                   **end if**12:                   ${\mathrm{confMap}}_{t_{i},p_{ID}}=\mathrm{Fuse}\left(motionMap,estimoteMap,t_{i},p_{ID}\right)$13:             **end for**14:        **end if**15:   **end if**16:   **if** $e_{i}.type=MOTION$ **then**17:          $e_{i}^{\left(L_{ms}\right)}=preProcess^{\left(L_{ms}\right)}\left(e_{i}\right)$            ▹ //$e_{i}^{\left(L_{ms}\right)}$ has a set of of fired sensors18:          **for** $p_{ID}$ in P **do**19:               ${\mathrm{confMap}}_{t_{i},p_{ID}}^{\left(L_{ms}\right)}=motionLocalizer\left(e_{i}^{\left(L_{ms}\right)}\right)$20:               $motionMap={\mathrm{confMap}}_{t_{i},p_{ID}}^{\left(L_{ms}\right)}$21:               $estimoteMap={\mathrm{confMap}}_{t_{i-1},p_{ID}}^{\left(L_{est}\right)}$22:               ${\mathrm{confMap}}_{t_{i},p_{ID}}=Fuse\left(motionMap,estimoteMap\right)$  ▹ //Produce final confMap for $p_{ID}$23:          **end for**24:     **end if**25:     PostProcess()                ▹ //Disambiguate for all persons in system26:**end procedure**

The Estimote Localizer produces the confidence map according to Algorithm 2. $t_{i}$ and $p_{ID}$ denote the timestamp of the incoming sensor event ($e_{i,p_{ID}}^{\left(L_{est}\right)}$) and the person whose movement caused the event. As mentioned above, the sensing area of the Estimote beacons and stickers, given the −70 dBm threshold, is approximated by a circle of diameter 1 m around each Estimote. The motion-sensor localizer generates a confidence map as described in Algorithm 3 every time it receives a new motion-sensor event, $e_{i}^{\left(L_{ms}\right)}$, containing information about all the motion sensors that fired in that timestamp.

**Algorithm 2** Estimote Localizer.1:**procedure** EstimoteLocalizer($e_{i,p_{ID}}^{\left(L_{est}\right)}$)2:     **for** all (x,y) **do**                        ▹ // All (x,y) in the area map3:          **if** $\left(x,y\right)\in SensingArea\left(e_{i,p_{ID}}^{\left(L_{est}\right)}.sensor_{beacon\_ID}\right)$ **then**  ▹ //If sensor in the event sees (x,y)4:             $m_{x,y}=1$5:          **else**6:             $m_{x,y}=0$7:          **end if**8:          $confMap_{t_{i},p_{ID}}^{\left(L_{est}\right)}\left(x,y\right)=m_{x,y}$            ▹ //Set confidence value for point (x,y)9:     **end for**10:**return** $confMap_{t,p}^{\left(L_{est}\right)}$11:**end procedure**

**Algorithm 3** Motion Localizer.1:**procedure** MotionLocalizer($e_{i}^{\left(L_{ms}\right)}$)2:     **for** all (x,y) **do**                      ▹ // All (x,y) in the area map3:          **for** all sensor in firedSensorSet **do**           ▹ firedSensorSet is inside $e_{i}^{\left(L_{ms}\right)}$4:               **if** (x,y)∈SensingArea(sensor) **then**5:                   $m_{x,y}=1$6:               **else**7:                   $m_{x,y}=0$8:              **end if**9:         **end for**10:        $confMap_{t_{i}}^{Motion}\left(x,y\right)=m_{x,y}$          ▹ //Set confidence value for point (x,y)11:   **end for**12:**return** $confMap_{t_{i}}^{Motion}$13:**end procedure**

### 3.3. Fusing Location Estimates

Depending on the algorithm it uses to compute its corresponding confidence-map for its location estimate, each localizer may use a different range of values in the representation of their confidence. In order to construct a common confidence value across all (two, in the case of this experiment) contributing localizers, the third step in the process involves a weighted summation of the input confidence maps, corresponding to each $p_{ID}$. When no eponymous sensors have been deployed in the space, the scheme reverts to a single confidence map that recognizes the likely locations for all individuals, without distinguishing among them.

The fusion step is simply the summation of the confidence values across the entire x,y-space using a weighted sum. Anonymous values are added to any eponymous values, but eponymous values can only be combined with the corresponding eponymous values (i.e., values for the same person $p_{ID}$). The weights are described in 4 as EstimoteReliability and MotionReliability. In this study, their values were set to 0.7 and 0.9, respectively; these values are based on the observed accuracy of the Estimote and motion sensors in our previous studies [1,12], which revealed that PIR motion sensors can be more accurate than Estimotes.

### 3.4. Outages and Outliers

As discussed above, the purpose of the fusion step is to merge the most recent location estimates produced by each distinct localizer into a single location estimate. However, either due to sensor malfunction or channel interference, one of the localizers may experience an “outage” (i.e., it may be silent for a long time). In this case, as new location estimates are produced by the other localizers, they will have to be fused with ever-older estimates. In this case, our method applies a confidence-reduction penalty to the fused estimate in order to recognize the fact that the two sources of evidence are out-of-sync and they may represent different states in the real world.

The function ReduceConfidence in Algorithm 4 receives as input the set of latest confidence maps from all localizers, and computes the time lapsed between the oldest and the most recent, $t_{diff}=t_{max}-t_{min}$. It returns as output the value of $\frac{1}{t_{dif}+0.2}$ as the confidence penalty, which is applied as a multiplier to each location in the fused confidence map.

**Algorithm 4** Location-Estimate Fusion.1:**procedure** Fuse(motionMap, estimoteMap, $t_{i}$, $p_{ID}$)2:     ER=EstimoteReliability3:     MR=MotionReliability4:     confMap=createemptymap5:     $\left[confidencePenalty^{\left(L_{est}\right)},confidencePenalty^{\left(L_{ms}\right)}\right]=ReduceConfidence\left(estimoteMap,motionMap\right)$          ▹ //The above line calculates confidence penalty for confMaps of $L_{est}$ and $L_{est}$6:     $lastLocationEstimate=getLastLocationEsimate(p_{ID})$    ▹ //Last location estimate for $p_{ID}$7:     **for** all(x,y) **do**8:          $estimoteConf=m_{t_{i},p_{ID}}^{\left(L_{est}\right)}\left(x,y\right)*ER$          ▹ // Multiply weights to $confMap^{\left(L_{est}\right)}$9:          $motionConf=m_{t_{i},p_{ID}}^{\left(L_{ms}\right)}\left(x,y\right)*MR$          ▹ // Multiply weights to $confMap^{\left(L_{ms}\right)}$10:         $confMap_{t_{i},p_{ID}}\left(x,y\right)=estimoteConf+motionConf$        ▹ //Sum the confidences11:         $distance=A^{*}.FindPathLength\left(lastLocationEstimate.location,\left(x,y\right)\right)$  ▹ //Find distance12:         $speed=\frac{distance}{t_{i}-lastLocationEstimate.time}$                   ▹ //Calculate speed13:         **if** speed>speedLimit **then**14:             ${\mathrm{confMap}}_{t_{i},p_{ID}}\left(x,y\right)*=speedPenalty$            ▹ //Multiply speed penalty15:        **end if**16:    **end for**17:**return** ${\mathrm{confMap}}_{t_{i},p_{ID}}$18:**end procedure**

Next, the method determines if the displacement by which the individual has potentially moved—from the last timestamp to the current one—is a potential “outlier”. This information is important for the purpose of rejecting the result of misfiring sensors that would result in placing the person at an unlikely distance from the previous location estimate. Let us call the weighted sum for person $p_{ID}$ at time $t_{i}$ as $s_{p_{ID},x,y}\left(t_{i}\right)$; then, the output location estimate (x,y) for $p_{ID}$ is the average of ${arg max}_{x,y\in Grid}s_{p_{ID},x,y}\left(t_{i}\right)$, where Grid are the square blocks in the area map.

The distance between successive location estimates is calculated with the help of an $A^{*}$ algorithm, using a finer location grid than the grid used for localization purposes. In the current configuration of our method, the $A^{*}$ algorithm runs on a 0.2 m grid, compared to the localization process which assumes a 1 m grid. The $A^{*}$ grid honors the spatial constraints imposed by the obstacles (i.e., not going through walls). The choice of a fine grid for the $A^{*}$ search is motivated by the need to capture features of the space that might hinder the movement of an individual. Using $A^{*}$ is motivated by our preference to include search algorithms that could work in a dynamic environment. Realistically, individuals and (some) obstacles may be moved (and tracked); therefore, spaces are dynamic enough to preclude the use of static shortest-path algorithms. A person’s potential speed is calculated based on the distance between the current and last location estimates and the time-difference between them; if the calculated speed is more than the normal speed of a walking person (approximately 1.3 m/s), the confidence of the cells in the new location estimate is reduced by a factor of 0.5; these steps are incorporated in Algorithm 4. The $A^{*}$ algorithm starts from the previous location estimate ${\left(x,y\right)}_{i-1,p_{ID}}$ at time $t_{i-1}$ and searches for the shortest path to the current location estimate ${\left(x,y\right)}_{i,p_{ID}}$. To speed up the process, at each point $po_{middle}$ in the middle of the search, as soon as the length of $path_{{\left(x,y\right)}_{i-1,p_{ID}}\to po_{middle}}$ plus the Euclidean distance between $po_{middle}$ and ${\left(x,y\right)}_{i,p_{ID}}$ is more than $\mathrm{normalspeed}\times (t_{i}-t_{i-1})$, the search stops and the confidence map is penalized. These steps are shown in lines 12–16 in Algorithm 4.

### 3.5. Disambiguation of Anonymous Persons

Let us consider the case where only motion sensors are utilized; in that case, the confidence maps produced will contain areas where the motion-sensor localizer identifies the potential presence of “some” individuals. In principle, the number for these areas will be less than or equal to the number of persons in the space: if two or more people congregate in roughly the same location, then there will be a single area corresponding to their presence as a group. This is exactly the scenario we investigated in [2].

If all individuals are also identified through an additional technology (such as in the case where all individuals are carrying smartphones and are recognized in the vicinity of Estimotes), then the sensor-fusion step results in merging the evidence collected from the various sensors in a single confidence map, where all areas are annotated with a person $p_{ID}$ to indicate some confidence for the presence of this specific person in the area.

There is yet another scenario: when one of the occupants is not tracked by anything other than the motion sensors; this situation may occur either because of an outage in Estimote data-transmission events, or because the occupant is not carrying any smartphone at all. This case happens in Figure 3, where person 1 carries a smartphone and is associated with the confidence map of Figure 3c, while person 2 is localized only by motion sensors and corresponds to the confidence map of Figure 3d. Then, it is possible to disambiguate the “anonymous” occupant (person 2 in Figure 3) with a post-processing step given that we have estimated where the other participant is located. This process results in Figure 3f for the second participant. This step involves a Gaussian mixture model (GMM) that treats the normalized confidence values of the confidence map as probabilities and clusters them. The GMM is not provided any information about the number of individuals present, and attempts to fit the best model possible [22]. In our example, in Figure 3d, the GMM returns two clusters, corresponding to the two dark red areas. Then, the confidence of the points in the cluster that has a distance smaller than 0.5 m to the areas that have been annotated with the $p_{ID}$s of the smartphone-carrying individuals (Figure 3c) is reduced, hence “subtracting” from the confidence values, and the remaining cluster in the confidence map, corresponding to the anonymous person (person 2) will have a higher probability of them being there, resulting in the confidence map at Figure 3f for person 2. This process is performed as the very last step of Algorithm 1.

## 4. Evaluation

Twenty-six participants were recruited to spend one two-hour shift—either alone or in pairs (seven pairs)—in the $SmartCondo^{\mathrm{TM}}$. The participants were asked to follow a scripted sequence of activities (i.e., an activity protocol). This protocol started with the subjects placing their personal belongings in the entrance closet; followed by performing some exercises in front of a Kinect; simulating personal-care activities including toileting and bathing; preparing a meal, eating it, and cleaning up; simulating doing laundry; playing some games on a tablet; and watching TV. Some activities were simulated (e.g., personal care, dressing) and others were real (e.g., cooking, ironing, exercising). For the two-participant sessions, the protocol was the same for both subjects, with the exception that the order of the activities was slightly modified, and that both participants were involved in the meal preparation and TV-watching activities. Each of the activities in the protocol was scripted in details as sequence of smaller tasks. For example, the instructions for the meal-preparation activity were to get the frying pan from cabinet, bring eggs from the fridge, get a spoon, stand in front of the kitchen island, cook scrambled eggs, etc. A tablet was provided to each participant, running an application that prompted them to perform the next step; when they were done with a specific task, they had to tap a “continue” button to go to the next task. In this manner, we can be sure that all the participants followed the exact same activity protocol. The participants were asked to wear an armband with a smart phone on their arm, either a Galaxy S4 or a Nexus 5 running Android 5, so that the smartphone was always with them and it did not interfere with their movement.

**Figure 4:**
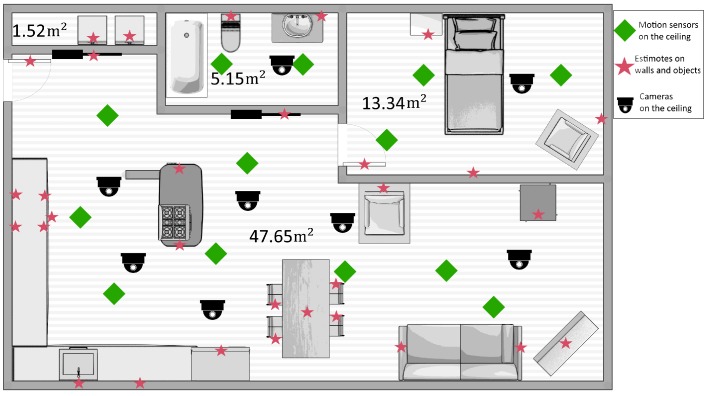
Layout of the $SmartCondo^{\mathrm{TM}}$ with positions of static Estimote stickers and PIR motion sensors.

A simplified floor plan of the $SmartCondo^{\mathrm{TM}}$ space is shown in Figure 4. The red stars indicate the locations of the Estimote stickers that were attached on static objects, which cost approximately $10 each. We also attached 12 Estimotes on movable objects used for the script activities, such as a cup, a frying pan, the garbage lid, etc. Moreover, 14 PIR motion sensors (built from scratch in our lab at a cost of $20–30 each) were installed on the ceiling, with a Raspberry Pi 3 (~$50) nearby to receive the motion sensor events and stream them to the server. The smartphone used costs approximately $150. The phone batteries last approximately 6–7 h when the accelerometer and magnetometer on the phone are used and the events are streamed to the server.

In keeping with the idea that the sensor-specific localizers can be selected from a wide range of offerings, the actual computational complexity introduced by our contribution is due to the fusion and post-processing steps. The fusion step involves the addition of the confidence values of different confidence maps produced by different localizers for each individual occupant. This addition takes place over a discretized grid. Hence, if we have “P” individuals, “L” localizers, and “B” grid points in the area, the complexity of the fusion step is O(P×L×B). Then, during the post-processing step, the process of confidence reduction for the grid points that are too far from the previous location estimates for each person is performed in O(P×B). Finally, the disambiguation of anonymous persons involves two phases. First, the confidence maps for each person are clustered together to determine the location-estimate areas (O(P×B)). Next, for each person “p” in the space, for each grid point “b” in the confidence map, the disambiguation method checks if “b” is inside another person’s location estimate area, and if so, the confidence of “b” is reduced; this last part can be done in $O(P^{2}\times B)$. As a result, the whole process is completed in $O(P\times L\times B+P^{2}\times B)$ and since the number of localizers is typically a small constant, decided a-priori and independent of P, the time complexity is essentially $O(P^{2}\times B)$. We remark that the generation of each localizer estimate reflected in *L* can be a significant overhead and varies among localizers.

### 4.1. Extracting the Ground Truth

To collect ground-truth data, the participants’ movements and actions were video-recorded by six cameras, also shown in the diagram of Figure 4. We subsequently analyzed these videos to annotate them with the ground truth, regarding the participants’ activities and locations.

The video annotation was performed manually by the first author, who reviewed the videos and recorded the locations of each participant at each point in time, similar to the process outlined in [23]. Although this procedure is bound to produce inaccuracies with respect to the precise timing of each activity and the exact location of the participants, it is currently the only methodological option, given the complexity of the activities and the maturity of current video-analysis methods.

To alleviate the complexity of the ground-truth video-annotation task, the experiment script given to participants included instructions for them to stand on marked locations on the floor while conducting specific tasks. Not surprisingly, our participants did not follow the instructions very precisely, and for most of the time, they were at unmarked locations for which we do not have exact [x,y] coordinates. During the video-annotation process, we estimated those coordinates based on their location relative to known landmark points around them. Another common source of error in manually establishing the ground truth is introduced when recording the participants’ locations while moving: those locations are semi-automatically estimated through interpolation between known timestamped locations.

Another problem with generating the ground truth was the fact that we had three (sometimes four) different sources of timestamps: (a) the smart phones carried by the participants, which send the Estimote events to the server; (b) the database timestamps of the motion-sensor data-transmission events; and (c) the video-recording timestamp. While all those clocks were synchronized at the level of timezone, date, hour, and minute, they were out of sync in terms of seconds. As a result, the timestamps of the ground truth and the inferred location and activity may differ from each other by as much as 60 s. To mitigate this problem, we use a time window of length T=1,30,60 s when determining the corresponding ground truth point for each of the system’s estimates at each time (see Equation (1)).

(1) $$Error_{t_{i}}=min_{t_{i}-T}^{t_{i}+T}∥l_{t_{i}}^{est}-l_{t_{i}}^{gt}\;$$

In the above equation, $l_{t_{i}}^{est}$ is the estimated location of an occupant at time $t_{i}$; $l_{t_{i}}^{gt}$ is the actual location of the same occupant at the same timestamp; and *T* is the window’s length.

### 4.2. Results

In this section, we examine the performance of our method. We report and discuss the location-estimate errors, calculated based on the formula in Equation (1), for single-participant sessions and two-participant sessions under different knowledge assumptions.

Table 1, Table 2, Table 3, Table 4 and Table 5 show our localization results for six of the 2-h sessions in our experiment: three of these sessions involved a single participant and the other three involved two participants. The name of the sessions used in all tables follows the convention “session1_i” to indicate the *i*-th single participant session. Similarly, “session2_i” is the *i*-th session with two participants.

Table 1 and Table 2 report the average error of our method in three single-participant sessions, under two different conditions: (a) using both motion sensors and Estimotes, and (b) using motion sensors only. Comparing the two tables, one notices the improvement in the localization accuracy that is possible due to the Estimotes. Estimotes improved the localization accuracy by approximately 20 cm on average. This is due to two reasons. First, the union of all the areas covered by the Estimotes is larger than the area covered by the motion sensors (Figure 4). Second, and more interestingly, the sensing area of each *individual* Estimote—given our −70 dBm threshold—is relatively smaller than that of the motion sensors, since they are mostly attached to the walls and detect targets within the semi-circle around them. Therefore, when the Estimotes recognize an occupant in their sensing area, they do so with high confidence, and the location estimate becomes more accurate. The standard deviation reported in Table 1 is higher than that of Table 2. This is because, unlike motion sensors, Estimote errors are not bounded; although we are assuming that RSSI values higher than −70 dBm imply that the target is within one meter of the Estimote, that may not be always the case. According to our previous study [12], the RSSI value can vary drastically (over a range of 10 dBm or more), even when the target is stationary at a fixed distance; this did not happen frequently in our experiment (so the accuracy is still better when adding Estimotes), but it is sufficient to make the standard deviation slightly higher (28 cm on average when window size was 1). In other words, the coverage area of the Estimote thresholded at −70 dBm is “fuzzy”. In contrast, a motion sensor firing means that the individual is within the (bounded) coverage area of the motion sensor.

Table 1 and Table 3 report the average error of our method in three single-participant sessions and three two-participant sessions, respectively. We are still in the process of annotating the remaining sessions of our experiment. It is easy to notice that our method exhibits the highest accuracy (and smallest error) when configured with a time-window of 60 s. Intuitively, the coarser the time granularity, the smaller the error. The average localization error when the window size is 60 s is somewhere between 0.38 m and 0.64 m (0.5 m on average based on the results from Table 1, Table 2, Table 3, Table 4 and Table 5), better than when a window size of 1 s was used. This fact shows the impact of unsynchronized sensor events on the quality of the method’s estimates. Our choice to use a 60-s window is well motivated by the fact that our data-emitting sensors and devices are not synchronized at the granularity of a second.

For the two-participant sessions, the location-estimation error when both participants wore a smartphone on their arm is reported in Table 3. Nevertheless, as we discussed above, we are interested in the performance of our method when only some of the participants carry smartphones. This is important for assisted-living facilities, where older adults are unwilling to wear any sensors or carry a smartphone but their caregivers typically have one. To simulate this scenario, we ran two different experiments for each session, ignoring the data emitted by one phone of a participant at a time, and we applied our location-estimation method to the remaining data to examine how effective our method’s disambiguation feature is in this reduced-knowledge condition. The average result from the two experiments for each session are reported in Table 4. Comparing the results between Table 3 and Table 4, we note a relatively small decline, which provides evidence for the robustness of our method. The participant who does not carry a phone—and as a result is not sensed by the Estimotes—is localized by the motion sensors only, which is possible because the sensing area of each motion sensor is larger than that of the Estimotes: motion sensors sense elements within a diamond around them with diameter of approximately 2 m, while the Estimotes—due to their on-wall placement—sense within a semicircle of 1 m radius.

For the two-person localization, to the best of our knowledge, all the previous studies required both subjects to wear some kind of sensor or tag, or to carry a smartphone. A 2013 study [3] reported 1.49 m accuracy, but because their method was device-less, it could not disambiguate the occupants. Making even more stringent hardware assumptions, a 2009 study [24] reported an error of 1 cm only, but required RF transmitters and receivers and assumed sensors wired to the transmitters carried by the occupants. In this scenario, the batteries of sensors mounted on different body parts lasted only about 1–2 h, and the coverage area of each transmitter was only 3 m and was sensitive to the presence of metal objects in the area. Clearly, even though the obtained error is quite impressive, the method cannot be applied in any real-world scenario.

In our experiment, we were able to achieve almost the same accuracy when only one of the two participants carried a smartphone (Table 3 and Table 4). When the space was equipped with motion sensors, our method was able to still infer the likely locations of the two participants and relies on the single source of eponymous data to disambiguate those locations.

Table 5 presents the errors obtained for the same two-participant sessions when only Estimotes were used, without taking any motion sensor data into account. Remarkably, the location estimate errors are better than those reported in Table 3 by approximately 10 cm. This is due to the larger area coverage resulting from the union of the individual covered areas by the deployed Estimotes—approximately 40 m${}^{2}$—compared to the 26 m${}^{2}$ collectively covered by the motion sensors.

In a simple mutation experiment, we eliminated every other Estimote sensor and recomputed the location-estimate error: the average error for both participants for session 2_3 became 3.24 m (for a time window of 60 s), which is worse than the result reported for the same session in Table 3 or Table 5. This confirms our intuition that the superior accuracy of the Estimotes-only location estimates is an artifact of their deployment density.

**Figure 5:**
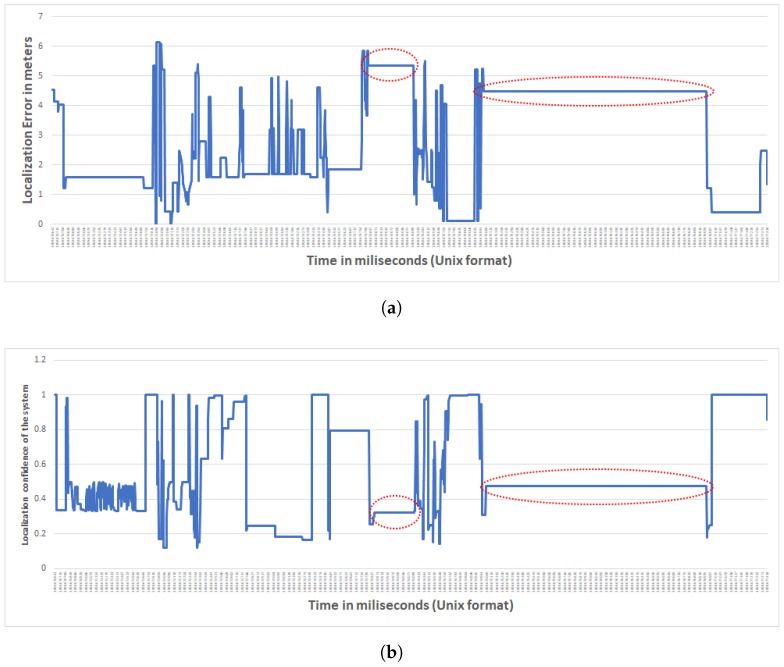
Figures (**a**) and (**b**) are from the same single occupant session. (**a**) Error of the localization for one of the single occupant sessions; (**b**) Confidence of the system on localizing one of the single occupant sessions.

Besides evaluating the accuracy of the location estimates produced by our method, we also analyzed its confidence. As we explained above, the output of the localization process is a confidence map for each person at each point in time, such as the one shown in Figure 3. Based on this confidence map, we also computed an overall confidence measure for the estimates produced by our method. As we discussed before, the confidence maps assign a value to each point in the monitored space, shown by colors in Figure 3: the deeper the color, the more confident our method is that there is someone—whether anonymous or eponymous—in that point. The overall confidence measure we discuss here is the maximum of these values over the whole map. Our hypothesis is that the high errors (more than 4 m) are due to prolonged periods without data. Indeed, there were time periods throughout the sessions during which the server did not receive events from our sensors despite the participants’ movements. The facility where the experiments were conducted is awash with RF interference, and possible network throughput deterioration and even outages are within the realm of possible. Indeed, our hypothesis on the origins of the inaccuracy and low overall confidence is validated in Figure 5. This Figure demonstrates that the method’s confidence is very low when the error is high, which implies that our method is “aware” of its blind spots. More precisely, when our method lacks input from sensors, based on Algorithm 1, the most recent confidence map is used but referring to Algorithm 4, the system reduces the confidence values. Hence, if the participant has been moving to a new location during the period for which the server did not receive any data-transmission events, the confidence would be low. In Figure 5 you can see this effect where in case of higher error for a long period of time (shown in the red-dotted areas in Figure 5a), the corresponding confidence measure is low (shown in the red-dotted areas in Figure 5b).

Finally, we conducted a preliminary analysis of our method’s effectiveness for activity recognition. There is a fairly limited set of activities that we are able to detect in our data. By attaching Estimotes on the objects shown in the left column of Table 6, we are able to recognize basic activities relying on the person’s interaction with the object in question. For example, when the Estimote attached on the iron is recognized by a participant’s smartphone, our method infers that the person is ironing. Several basic activities are grouped under a single “activity” header. For example, using an iron or a laundry basket or a washer or a dryer implies that the person is “doing laundry”. During all of the abovementioned sessions, our method was able to correctly detect 70% of the activities that the occupants were doing.

## 5. Conclusions

In this study, we have addressed the problem of estimating the location of multiple individuals moving and interacting in an indoor space. Our work makes two key contributions. First, it proposes a multi-sensor data-fusion framework, relying on a unifying location-estimate representation as a *confidence map* of the indoor space. In this framework, each distinct type of sensor data is processed by a sensor-specific algorithm to generate a sensor-specific *confidence map*; all sensor-specific confidence maps are subsequently fused into a single set of confidence maps corresponding to location estimates for each individual. Second, our framework distinguishes between anonymous and eponymous sensors, such as motion sensors and Estimote stickers. This combination enables our method to accurately recognize individuals when all, or all except one, carry a smartphone running the app that collects the Estimote sensor events. The “all except one” scenario is extremely important because it is motivated by the requirements of real settings involving caregivers who are willing to adopt and carry technologies such as smartphones, but the cared-for person is unable or unwilling to consistently use such a device. Table 7 compares our method and recent related work in this area. The Table demonstrates that our method makes realistic knowledge assumptions, does not require onerous configuration-deployment effort, and has been thoroughly evaluated on realistic scenarios.

We conducted six experiments, involving data collection from a real environment. We established that, using our method, the location of the individual that is not carrying/wearing a device on them can be determined just as accurately as it would have been if the individual was carrying a device. We also identified several crucial parameters that influence the accuracy of the proposed scheme. One of them is the relative coverage of the space by the sensing “footprint” of the eponymous data collected via the Estimotes: the larger this space is, and the higher the number of Estimotes deployed, the smaller is the impact of the coverage by the anonymous (motion) sensors. Nevertheless, this has to be seen against the backdrop of deciding on an RSSI threshold for the Estimote signals (−70 dBm in this study), which effectively transforms the Estimotes into proximity sensors. We also noticed that the lack of synchronization across the two (and potentially more) sources of sensed data, if not addressed at a lower layer, has to be accommodated when defining the accuracy metrics of a “fused” location-estimation scheme. By affording a 60 s window delay, we could derive more accurate location estimations than for shorter delays. The situation may be quite typical in future systems that use completely heterogeneous sources of data, utilizing different technologies and communication standards. In most such cases, no single one-size-fits-all low-level synchronization solution can be used, and the onus of synchronization shifts to the application layer.

We have conducted and reported on a number of experiments demonstrating the efficacy of our method, but we also note that a significant cost of the overall endeavor was the ground-truth annotation of the source data. To the extent that one high-priority item that impedes future research can be described, it will have to be the ability to automatically (or at least semi-automatically) annotate ground truth from captured traces. The use of computer-vision techniques may be indispensable for such a task.

Finally, while we did not make any deliberate attempt to optimize the efficiency of the algorithm implementations we used, we note that our method delivers an almost real-time behavior, taking an average of two seconds of execution time for each new location estimation. However, further analysis of the performance aspect is necessary—especially given its dependence on the size of the monitored space due to the grid-level discretization. Based on our observations during this study, we believe that the battery lifetime can be improved further by sending a “stop” message from the server to the phone, to stop scanning for and streaming Estimote data when the caregiver’s phone is far away from the area where the cared-for person may be located.This condition can be identified when the Google geolocation API localizes the device away from the residence of the patient and no relevant Estimote readings have been received for some time. Another frequently employed technique is to identify from accelerometer data that the phone or smartwatch is stationary and to throttle or stop sending updates, but such inactivity might mean that the individual has forgotten or not worn the device, and the protocol to react to such exceptions is dependent on the exact context. This feature—namely, controlling the phone application operation for the sake of extending its battery life—will be the subject of our future work.

## Figures and Tables

**Table 1:** Localization error for single-participant sessions, using motion-sensor and Bluetooth Low-Energy (BLE)-Estimote data. All the measurements are in meters.

Window Size	Session 1_1		Session 1_2		Session 1_3	
	Mean	Std Dev	Mean	Std Dev	Mean	Std Dev
1 s	1.92	1.34	2.35	1.80	2.88	1.72
30 s	1.52	1.09	1.88	1.53	2.59	1.73
60 s	1.31	0.86	1.71	1.39	2.50	1.72

**Table 2:** Localization error for single-participant sessions, using motion-sensor data only. All the measurements are in meters.

Window Size	Session 1_1		Session 1_2		Session 1_3	
	Mean	Std Dev	Mean	Std Dev	Mean	Std Dev
1 s	2.28	1.35	2.31	1.54	3.28	1.14
30 s	1.79	1.01	1.77	1.19	3.01	1.45
60 s	1.58	0.73	1.60	1.05	2.93	1.45

**Table 3:** Localization error for two-participant sessions, using motion-sensor and BLE-Estimote data, with both participants holding phones. All the measurements are in meters.

Window Size	Session 2_1		Session 2_2		Session 2_3	
	Mean	Std Dev	Mean	Std Dev	Mean	Std Dev
1 s	2.42	1.43	2.39	1.70	2.17	1.80
30 s	2.01	1.19	2.11	1.57	1.82	1.53
60 s	1.87	1.11	2.00	1.51	1.65	1.37

**Table 4:** Localization error for two-participant sessions, using motion-sensor and BLE-Estimote data with only one participant holding a phone. All the measurements are in meters.

Window Size	Session 2_1		Session 2_2		Session 2_3	
	Mean	Std Dev	Mean	Std Dev	Mean	Std Dev
1 s	2.53	1.43	2.49	1.83	2.33	1.77
30 s	2.06	1.00	2.22	1.73	1.94	1.52
60 s	1.92	0.90	2.1	1.67	1.77	1.35

**Table 5:** Localization error for two-participant sessions, using BLE-Estimote data only, with both participants holding phones. All the measurements are in meters.

Window Size	Session 2_1		Session 2_2		Session 2_3	
	Mean	Std Dev	Mean	Std Dev	Mean	Std Dev
1 s	2.31	1.26	2.38	1.49	1.91	1.36
30 s	1.92	1.08	2.12	1.38	1.61	1.07
60 s	1.81	1.06	1.98	1.34	1.46	0.97

**Table 6:** Activities recognized based on Estimotes attached to objects.

Basic Activities	Activities
Use iron, Use ironing board, Move laundry basket, Use dryer	Laundry
Use dustpan, Use broom	Brooming
Use TV remote	Watching TV
Use kettle, Use frying pan, Use cup, Open/Close kitchen cabinet Open/Close fridge, Open/Close garbage lid	Cooking/Eating/Washing Dishes
Take medication	Medication

**Table 7:** Comparing indoor localization methods.

	Our Method	Ruan [5]	Chen [7]	Torres [10]	Zou [11]	Xu [3]	Galatas [20]	Mohebbi [12]	Azghandi [2]	Vlasenko [1]
Uses wearables	yes	yes	yes	yes	yes	no	yes	yes	yes	no
Needs sensor location	yes	yes	yes	yes	yes	yes	yes	yes	yes	yes
Requires training, fingerprinting, model fitting	no	yes	yes	yes	yes	yes	no	yes	no	no
Number of test scenarios with people	6	3	2	1	1	1	2	3	0	0
Length of each experiment	120 min	1 min	NR	NR	NR	NR	NR	30–60 min	NR	NR
Natural movements during experiment	yes	yes	no	no	no	no	yes	yes	simulation	simulation
Mean error of single person localization	1.8	0.58	1.28–1.39	2	0.59	1.3	NR	1.8	NR	0.4–1.4
Mean error of multi-person localization	1.8	NR	NR	NR	NR	NR	67–87% detection in room	NR	0.6–1.6	NR

${}^{1}$ Best method at the IPIN 2016, HFTS Team; ${}^{2}$ window size = 60 s; ${}^{3}$ Not Reported.
